# Insights into the mechanism of cyanobacteria removal by the algicidal fungi *Bjerkandera adusta* and *Trametes versicolor*

**DOI:** 10.1002/mbo3.1042

**Published:** 2020-06-11

**Authors:** Guomin Han, Hui Ma, Shenrong Ren, Xueyan Gao, Xiaolong He, Suwen Zhu, Ruining Deng, Shihua Zhang

**Affiliations:** ^1^ School of Life Sciences Anhui Agricultural University Hefei China; ^2^ National Engineering Laboratory of Crop Stress Resistance Breeding Anhui Agricultural University Hefei China; ^3^ Key Laboratory of Rice Genetic Breeding of Anhui Province Rice Research Institute Anhui Academy of Agricultural Sciences Hefei China; ^4^ Institute of Applied Mathematics Anhui Agricultural University Hefei China

**Keywords:** Algicidal fungi, Algicidal mechanism, Decomposition, Endopeptidase, Polysaccharide lyases8, Transcriptomic analysis

## Abstract

Fungal mycelia can eliminate almost all cocultured cyanobacterial cells within a short time. However, molecular mechanisms of algicidal fungi are poorly understood. In this study, a time‐course transcriptomic analysis of algicidal fungus *Bjerkandera adusta* T1 was applied to investigate gene expression and regulation. A total of 132, 300, 422, and 823 differentially expressed genes (DEGs) were identified at 6, 12, 24, and 48 hr, respectively. Most DEGs exhibited high endopeptidase activity, cellulose catabolic process, and transmembrane transporter activity by using Gene Ontology (GO) and Kyoto Encyclopedia of Genes and Genomes (KEGG) analyses. Many decomposition genes encoding endopeptidases were induced a little later in *B. adusta* T1 when compared with previously investigated algicidal fungus *Trametes versicolor* F21a. Besides, the accumulated expression of Polysaccharide lyases8 (PL8) gene with peptidoglycan and alginate decomposition abilities was greatly delayed in *B. adusta* T1 relative to *T. versicolor* F21a. It was implied that endopeptidases and enzymes of PL8 might be responsible for the strong algicidal ability of *B. adusta* T1 as well as *T. versicolor* F21a.

## Graphical abstract

Algicidal process of fungus *Bjerkandera adusta* T1 was investigated by a time‐course transcriptomic analysis. The results showed that most differentially expressed genes exhibited high endopeptidase activity, cellulose catabolic process, and transmembrane transporter activity. Comparison of gene expression between *B. adusta* T1 and Trametes versicolor F21a during the algicidal process suggested that endopeptidases together with enzymes of the polysaccharide lyases family 8 might be responsible for the strong algicidal activity of *B. adusta* T1 as well as T. versicolor F21a.

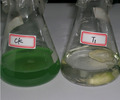

## 1 INTRODUCTION

The occurrence of algal blooms or cyanobacterial blooms not only leads to the asphyxiation of aquatic fauna, but also releases highly toxic compounds, including microcystins, threatening the health of human beings and other organisms (Dai et al., 2018; Sun, Sun, Zhang, Esquivel‐Elizondo, & Wu, 2018). Biological methods are known to be simple and efficient to control algal blooms, with less pollution compared with the physical and chemical methods (Hou et al., 2019; Yu et al., 2019; Zhang et al., 2018). In addition to the inhibition of cyanobacterial growth, algicidal bacteria and viruses can affect the water clarity and aquatic ecosystem (Wang et al., 2010). Recently, a new method for the removal of cyanobacteria by fungi was reported (Jia et al., 2010). Further, it has been reported that the mycelia of fungus *Trichaptumabietinum* 1302BG could enclose and eliminate almost all cocultivated cyanobacterial cells within a short time (Jia et al., 2010), and the color of cyanobacterial medium turned transparent (Han et al., 2011). Other fungi, such as *Trametes versicolor* F21a, *Bjerkandera adusta* T1, *Lophariaspadicea*, *Phanerochaete chrysosporium*, *Trichoderma citrinoviride*, and *Irpexlacteus* T2b have been reported to exhibit algicidal ability (Han et al., 2011; Shu et al., 2016; Wang et al., 2010; Zeng, Wang, & Wang, 2015; Zeng et al., 2019). Among these, *T. versicolor* F21a and *B. adusta* T1 were considered as the two best algicidal fungi (Dai et al., 2018; Han et al., 2011; Zeng et al., 2015, 2019).

Previous studies have reported that both living and dead cyanobacterial cells first adhere to fungal mycelia before being eliminated by surrounding mycelia (Dai et al., 2018; Jia et al., 2010). It has been further demonstrated that the membranes of cyanobacterial cells and the pyrrole ring of chlorophyll *a* were extensively disrupted by mycelia of *P. chrysosporium* (Zeng et al., 2015). Transcriptomic and proteomic analyses of the algicidal mechanism of *T. versicolor* F21a showed that several biological processes, such as glucan 1,4‐α‐glucosidase activity, hydrolase activity, lipase activity, and endopeptidase activity, and Kyoto Encyclopedia of Genes and Genomes (KEGG) pathways, including glycolysis/gluconeogenesis, pyruvate metabolism, starch and sucrose metabolism, and amino acids biosynthesis, are involved in the elimination cyanobacterial cells (Dai et al., 2018; Gao et al., 2017). The expression of all Carbohydrate‐Active enZYmes (CAZyme) genes significantly increased during the algicidal process in *T. versicolor* F21a (Dai et al., 2018; Gao et al., 2017). Several members of CAZyme, such as AA5, GH18, GH5, GH79, GH128, and PL8, might play key roles in the decomposition of cyanobacterial cells at different eliminating stages (Dai et al., 2018). Although the underlying molecular mechanism of algicidal fungus *T. versicolor* F21a was elucidated, there are no reports on the mechanism of other efficient algicidal fungi.

*B. adusta* is a widely distributed “white rot” fungus, which has been often associated with the decomposition of hardwoods (Moody, Dudley, Hiscox, Boddy, & Eastwood, 2018). The components of wood cell walls, such as cellulose, hemicellulose, and recalcitrant lignin, can be degraded by this fungus (Moody et al., 2018). Besides, this fungus has been reported to decompose a wide range of environmental pollutants (Bouacem et al., 2018; Han et al., 2011; Sugawara, Igeta, Amano, Hyuga, & Sugano, 2019). In our previous study, *B. adusta* T1 was found to be one of the best algicidal fungi (Han et al., 2011). In this study, gene expression in the mycelia of *B. adusta* T1, cocultivated with and without cyanobacterial cells during the algicidal process, was compared by a time‐serial transcriptomic analysis. Differentially expressed genes (DEGs) were used to identify key decomposition gene(s) and pathway(s) in *B. adusta* T1, and the results were compared with that of *T. versicolor* F21a reported in a previous study (Dai et al., 2018).

## 2 MATERIALS AND METHODS

### 2.1 Fungal and algal strains

The previously isolated fungus *B. adusta* T1 from Zijinshan Mountain was used in this study (Han et al., 2011). Cyanobacterial strain (*Microcystis aeruginosa* PCC7806) was provided by the Institute of Hydrobiology of the Chinese Academy of Sciences (Wuhan, China).

### 2.2 Cocultivation of fungal mycelia and cyanobacterial cells

The cyanobacterial strain was cultivated at 25°C under 12‐hr light and 12‐hr dark cycles with ~90 μmol/m^2^ s^‐1^ of photons in BG‐11 medium (Jia et al., 2010). Round fungal mycelium (seven mm in diameter) was inoculated onto a nine‐cm plate, containing 15 ml of potato liquid medium, and incubated under static conditions for five days. Then, fungal mycelia were taken and transferred into 250‐mL Erlenmeyer flasks containing 100 ml of algal solution or medium. The cocultures were incubated at 25°C, 90 μmol photons/m^2^ s^‐1^, and 120 rpm to investigate differentially expressed fungal genes. Total chlorophyll *a* was measured according to the Standard Methods for the Examination of Water and Wastewater (Standard Methods for the Examination of Water & Wastewater, 1998).

### 2.3 RNA isolation and sequencing

Mycelia of *B. adusta* T1 were collected from cocultures after 6, 12, 24, and 48 hr of incubation. Two biological replicates of each treatment were used for RNA sequencing. Total RNA was extracted from each sample with TRIzol reagent following the manufacturer's instructions (Takara, Dalian, China). Then, crude RNA was digested via 10 U DNase I (TaKaRa, Japan) at 37°C for 30 min, and then, mRNA was isolated using Dynabeads® Oligo (dT) 25 (Life, America) following the manufacturer's instructions. One hundred ng mRNA of each sample was used to construct a sequencing library using NEBNext® Ultra^TM^ RNA Library Prep Kit (NEB, America). Paired‐end sequencing of cDNA fragments (~300 bp) was performed using Illumina HiSeq 4,000 platform at BGI‐Shenzhen, China.

### 2.4 Transcriptomic analysis

In this study, RNA‐Seq data of *B. adusta* T1 at 6, 12, 24, and 48 hr were analyzed. The quality of 150‐bp reads was assessed using the FASTQC program (http://www.bioinformatics.babraham.ac.uk/projects/fastqc/). The paired‐end raw reads from RNA sequencing were trimmed using the pipeline Trimmomatic (v0.33) with parameters (LEADING:3 TRAILING:3 SLIDINGWINDOW:4:15 HEADCROP:12 MINLEN:36) (Bolger, Lohse, & Usadel, 2014). The clean reads were mapped to the *B. adusta* genome (v1.0) using STAR software (v2.5.3a) (Binder et al., 2013; Dobin et al., 2013). Expression value in FPKM (fragments per kilobase of exon model per million reads mapped) and DEGs were calculated via Cuffdiff (v2.2.1) using default parameters (*p* < .05, a fold change ≥ 2) (Si et al., 2019; Trapnell et al., 2012). Gene function was annotated using BLAST against reference protein‐encoding sequences from the Nr database of GenBank, Gene Ontology (GO), and KEGG (Ashburner et al., 2000; Kanehisa, Furumichi, Tanabe, Sato, & Morishima, 2017; Kanehisa & Goto, 2000; Kanehisa, Sato, Kawashima, Furumichi, & Tanabe, 2016). Fisher's exact test was used to obtain enriched functional terms at *p* < .05.

### 2.5 CAZyme and Secretome Annotation

All putative protein sequences of *B. adusta* were annotated with hmmscan against dbCAN database (Cantarel et al., 2009; Johnson, Eddy, & Portugaly, 2010; Yin et al., 2012) and further classified according to mycoCLAP database (Strasser et al., 2015). Signal information of the proteins was predicted by Target P 1.1 Server (Emanuelsson, Brunak, von Heijne, & Nielsen, 2007).

### 2.6 Quantitative PCR (qPCR) validation

qPCR was used to validate the gene expression calculated from RNA‐Seq data. A few randomly selected lignocellulose‐active enzyme genes were used in this study, and the β‐actin gene of *B. adusta* T1 was used as the endogenous control. The 20 μl reaction mixture consisted of 10 μl SYBR® Fast qPCR Mix (2x), 0.5 μl of each primer (10 μmolL^−1^), and 120–150 ng cDNA (Table A1). The qRT‐PCR program was set as follows: 95°C for 10 min, followed by 40 cycles of 95°C for 15 s, 60°C for 20 s, and 72°C for 30 s. Relative expression levels were calculated using 2^−ΔΔ^CT method (Livak & Schmittgen, 2001). Three biological replicates were used for qRT‐PCR.

## 3 RESULTS

### 3.1 Elimination rate during the algicidal process

**FIGURE 1:**
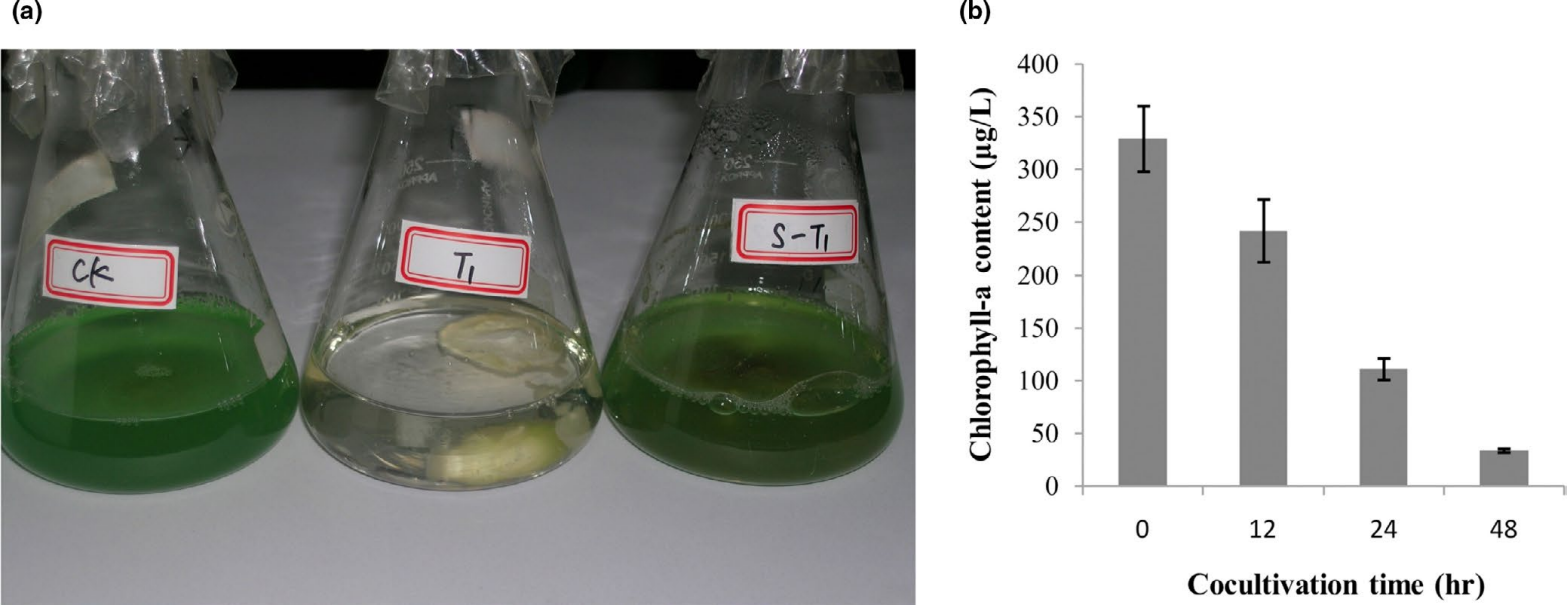
Changes in the algicidal process of *B. adusta* T1. Note: (a) Images of cocultivation after 48 hr; CK, the cyanobacterial cells as control;T1, the cocultivation of cyanobacterial cells and *B. adusta* T1 mycelia; S‐T1, the cocultivation of cyanobacterial cells and died fungal mycelia. (b) Changes in chlorophyll *a* content during the algicidal process

The algicidal process of *B. adusta* T1 was monitored via spectrophotometer. As shown in Figure 1, the chlorophyll a content gradually decreased with the increase in incubation time. Approximately 86% of cyanobacterial cells were eliminated within 48 hr. The cyanobacterial cells were almost disappeared in the flask cocultivated with living fungal mycelia while the cyanobacterial cells were almost not affected by dead fungal mycelia compared with the blank control (Figure 1).

### 3.2 RNA‐Seq data generation and mapping

**FIGURE A1:**
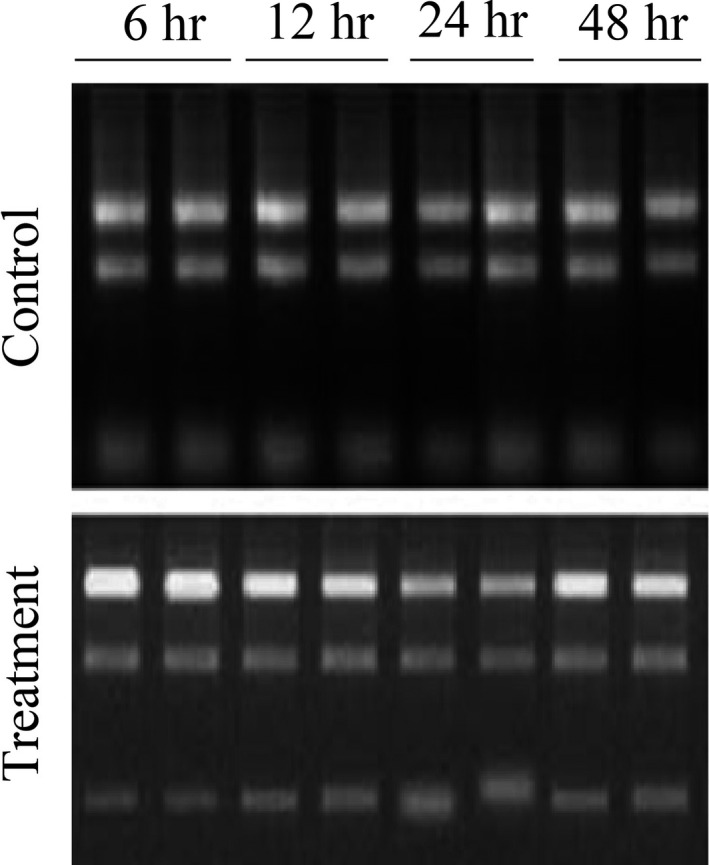
Total RNAs extracted from mycelia co‐cultivated with cyanobacterial cells (Treatment) and without cyanobacterial cells (Control) of 6, 12, 24, and 48 hr samples

Mycelia of *B. adusta* T1 that was cocultivated with cyanobacterial cells at 6, 12, 24, and 48 hr were used for RNA sequencing. Fungal mycelia without cyanobacterial cells at the same time point were used as a control. Good quality RNA was isolated and used for RNA sequencing (Figure A1). A total of 63,437,015 pairs of raw reads (SRA accession: PRJNA543936) were generated (Table A2). Approximately 96% of reads were retained after the removal of adaptor and low‐quality bases (Table A2). More than 64% of reads were uniquely mapped to the reference genome by pipeline STAR (Table A2), suggesting that the results of mapping can be used for the identification of fungal DEGs.

### 3.3 Identification of fungal DEGs involved in the algicidal process

**FIGURE 2:**
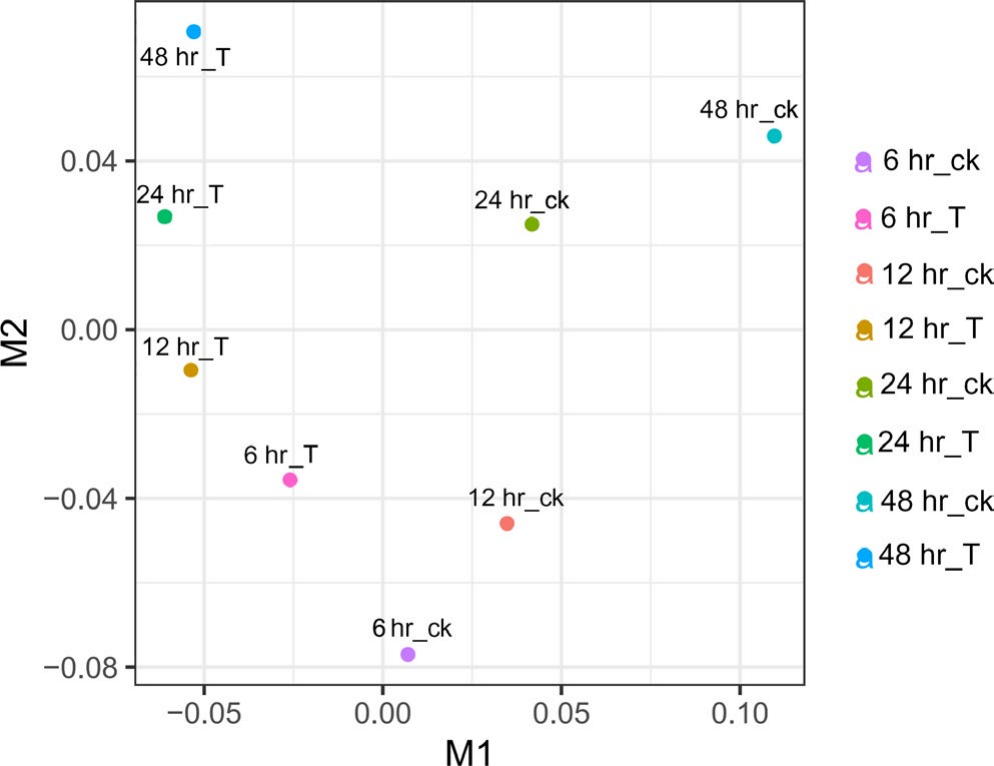
Multi‐dimensional scaling of gene expression data. Note: 6h_ck, control sample at 6h; 6h_T, treatment sample at 6 hr; 12h_ck, control sample at 12 hr; 12h_T, treatment sample at 12 hr; 24h_ck, control sample at 24 hr; 24h_T, treatment sample at 24 hr; 48h_ck, control sample at 48 hr; 48h_T, treatment sample at 48 hr

**FIGURE 3:**
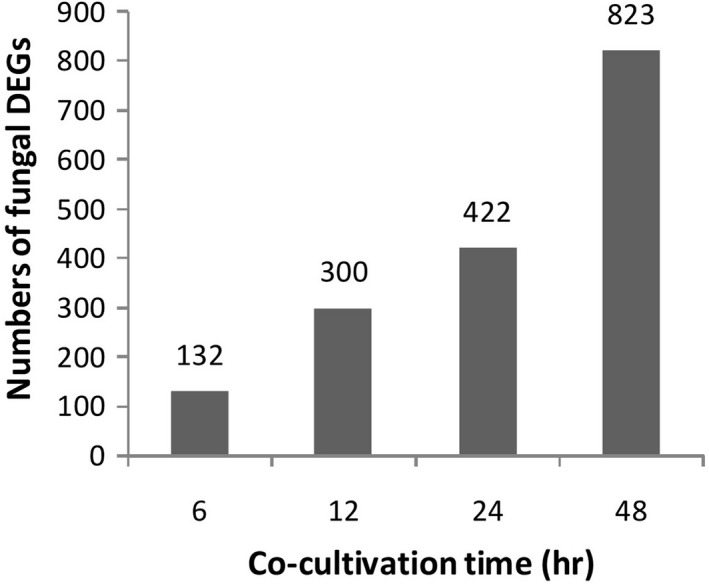
Number of fungal DEGs during the algicidal process of *B. adusta* T1

**FIGURE A2:**
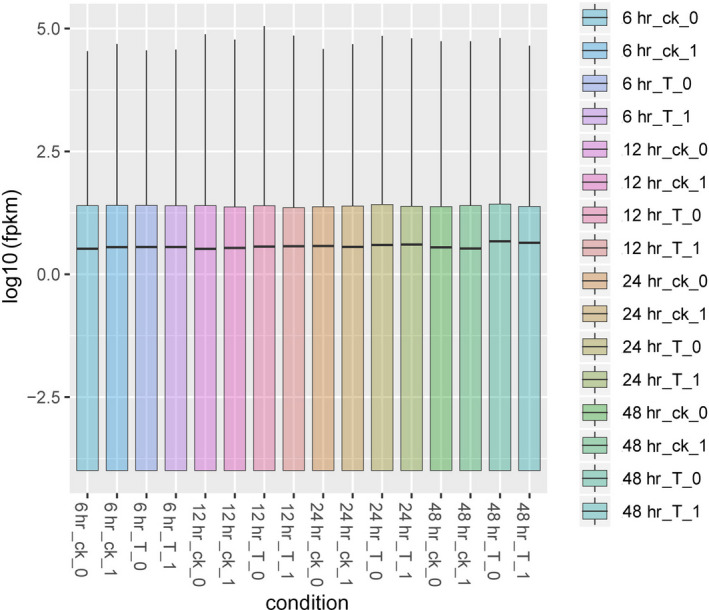
Boxplots showing the distribution of the FPKM values of each sample. Note: 6h_ck, control sample at 6 hr; 6h_T, treatment sample at 6 hr; 12h_ck, control sample at 12 hr; 12h_T, treatment sample at 12 hr; 24h_ck, control sample at 24 hr; 24h_T, treatment sample at 24 hr; 48h_ck, control sample at 48 hr; 48h_T, treatment sample at 48 hr. “_0” and “_1” represent repeat samples

**FIGURE A3:**
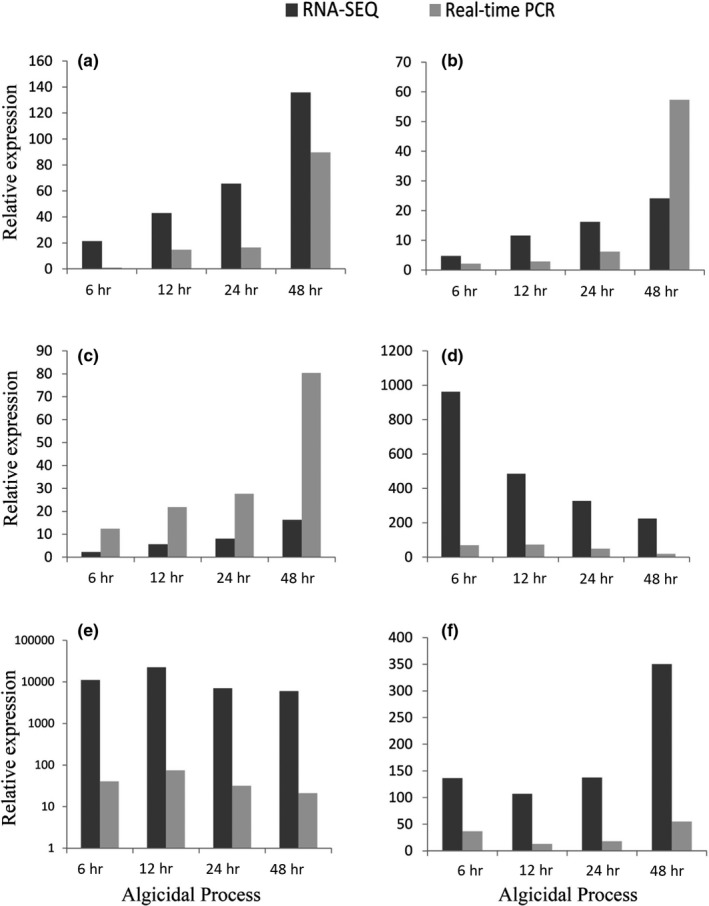
Comparison of expression changes between Real‐time PCR and RNA‐Sequencing. Note: A, jgi|Bjead1_1|34143|fgenesh1_kg.1_#_945_#_Locus732v1_medCvg1115.6s (a protein of esterase family 1); B, jgi|Bjead1_1|35099|fgenesh1_kg.2_#_711_#_Locus118v3_medCvg9284.2s (a protein of hydrolase family 5); C, jgi|Bjead1_1|172436|gm1.8875_g (a protein of hydrolase family 5); D, jgi|Bjead1_1|459664|MIX10988_17319_14 (a radical oxidase); E, jgi|Bjead1_1|355947|CE167616_517 (a protein of hydrolase family 128); F, jgi|Bjead1_1|38229|fgenesh1_kg.7_#_551_#_Locus8080v1_medCvg1578.8s (a protein of hydrolase family 13)

Boxplot of FPKM values across all samples showed the consistency of biological replicates of each treatment (Figure A2). Multi‐dimensional scaling (MDS) showed that the gene expression in mycelia cocultured with cyanobacterial cells was distinctly separated from that of mycelia without cyanobacterial cells (Figure 2). The difference became highly apparent with the increase in cocultivation time (Figure 2). A total of 132, 300, 422, and 823 fungal DEGs were identified at 6, 12, 24, and 48 hr in the mycelia cocultivated with cyanobacterial cells compared with the control, respectively (Figure 3). The expression of six randomly selected lignocellulose‐active enzyme genes, that is*,* a gene of esterase family, two genes of hydrolase family, a gene of hydrolase family 5, a radical oxidase encoding gene, a gene of hydrolase family 128, and a gene of hydrolase family 13, were further investigated via qRT‐PCR (Table A1). Similar expression patterns were observed between qRT‐PCR and transcriptomic analysis (Figure A3), indicating that DEGs identified by the transcriptomic analysis were suitable for further analyses.

### 3.4 Annotation and enrichment analyses of fungal DEGs

**FIGURE 4:**
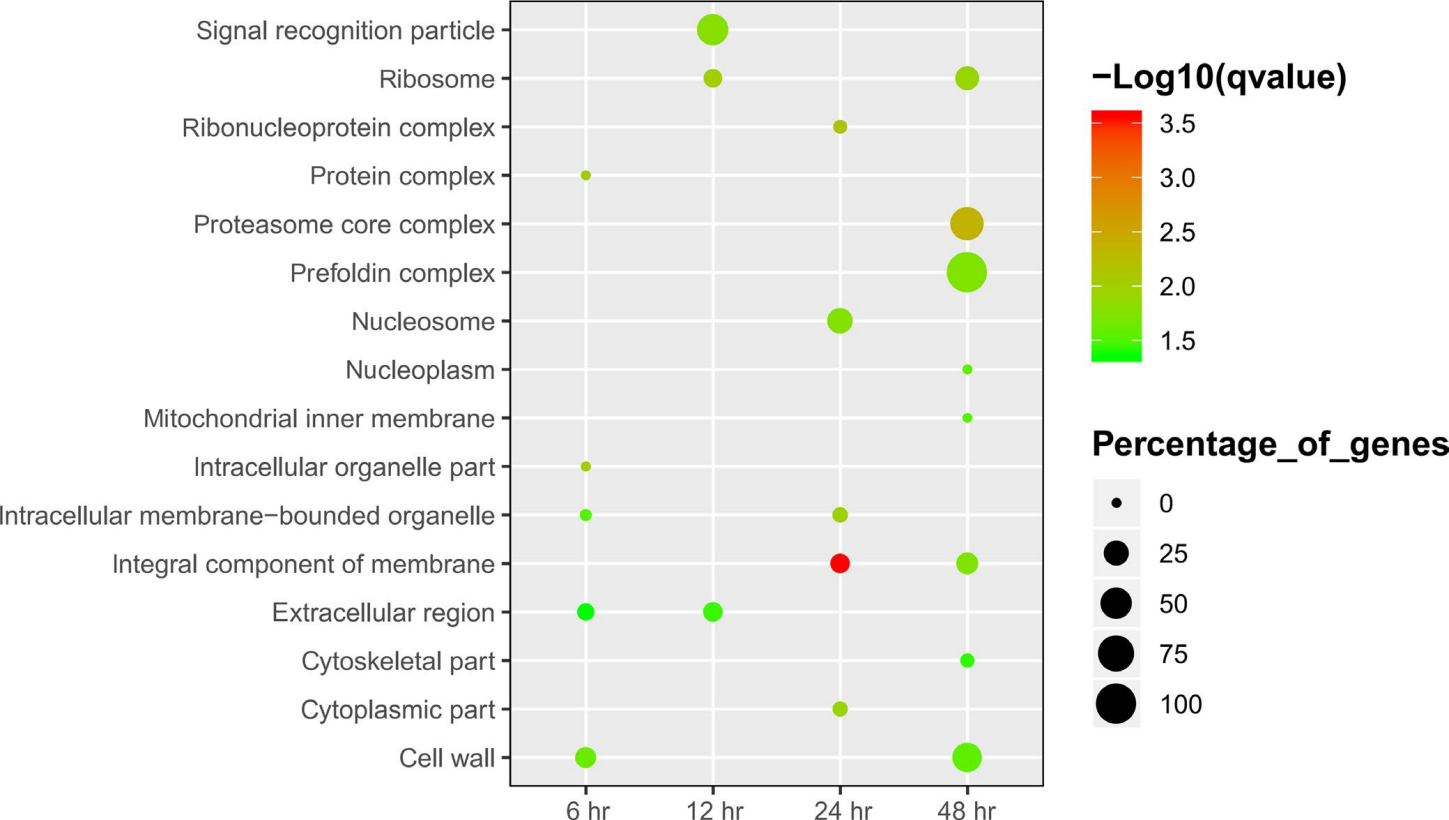
GO term enrichment of fungal DEGs in the cellular component category

**FIGURE 5:**
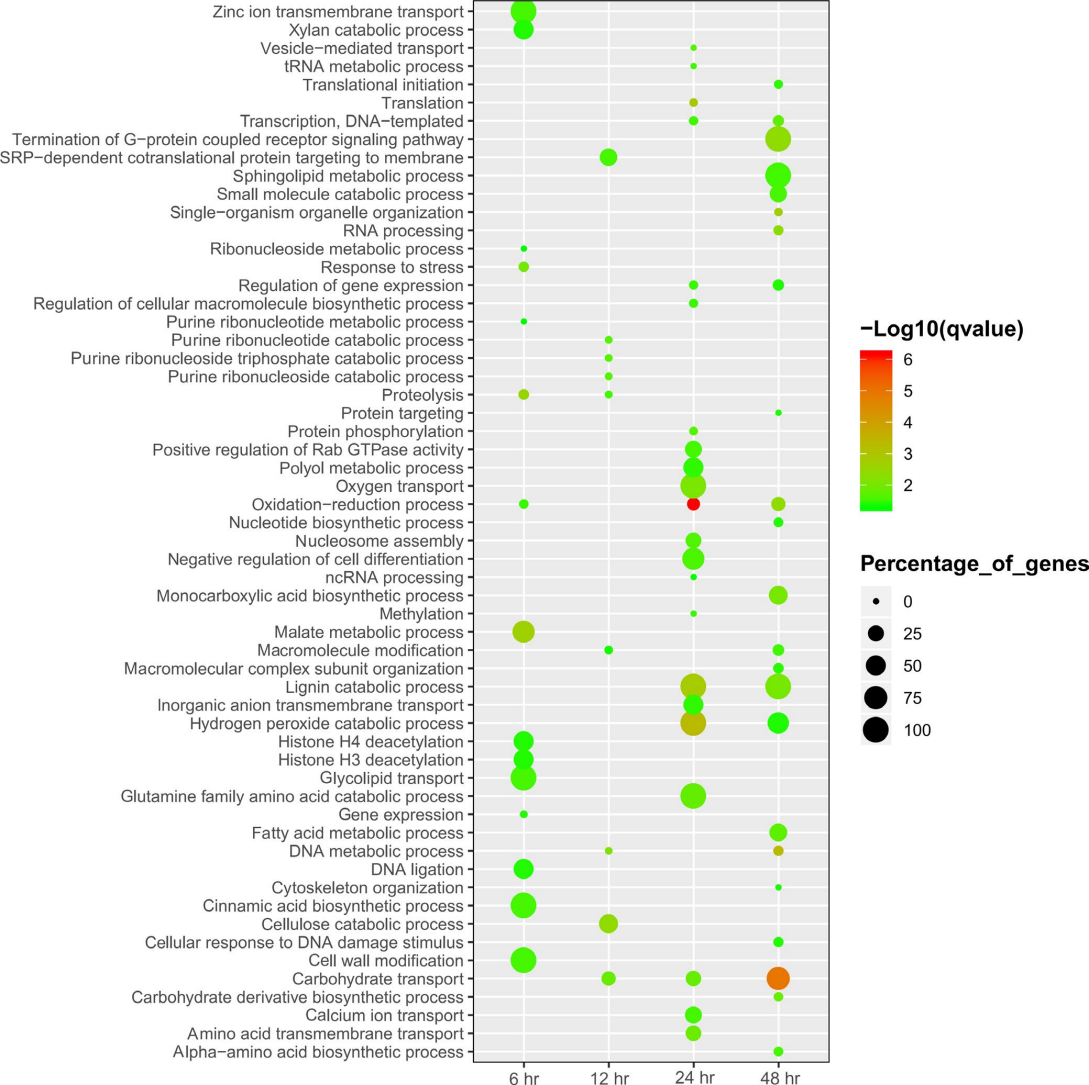
GO term enrichments of fungal DEGs in the biological process category

**FIGURE 6:**
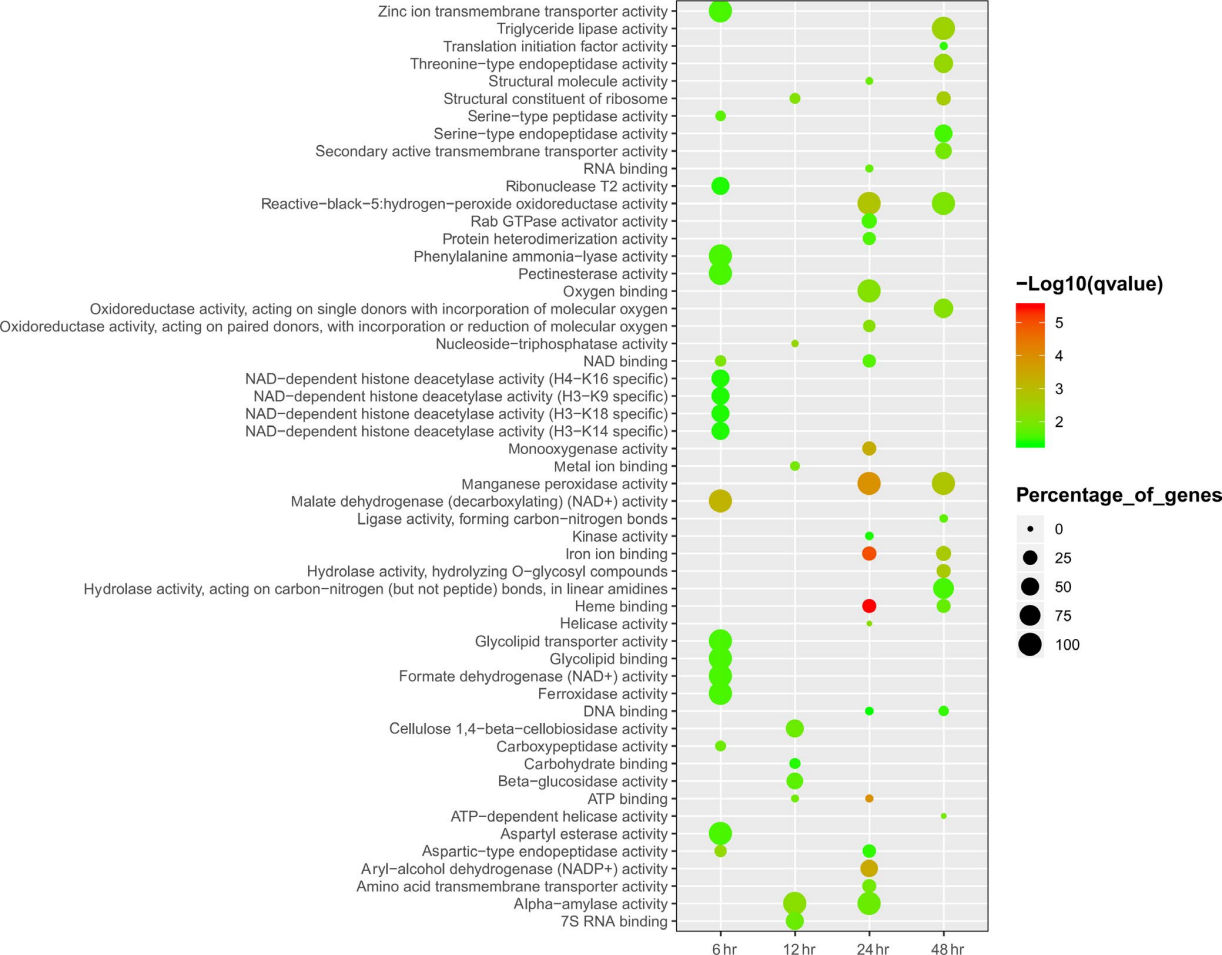
GO term enrichments of fungal DEGs in the molecular function category

**FIGURE 7:**
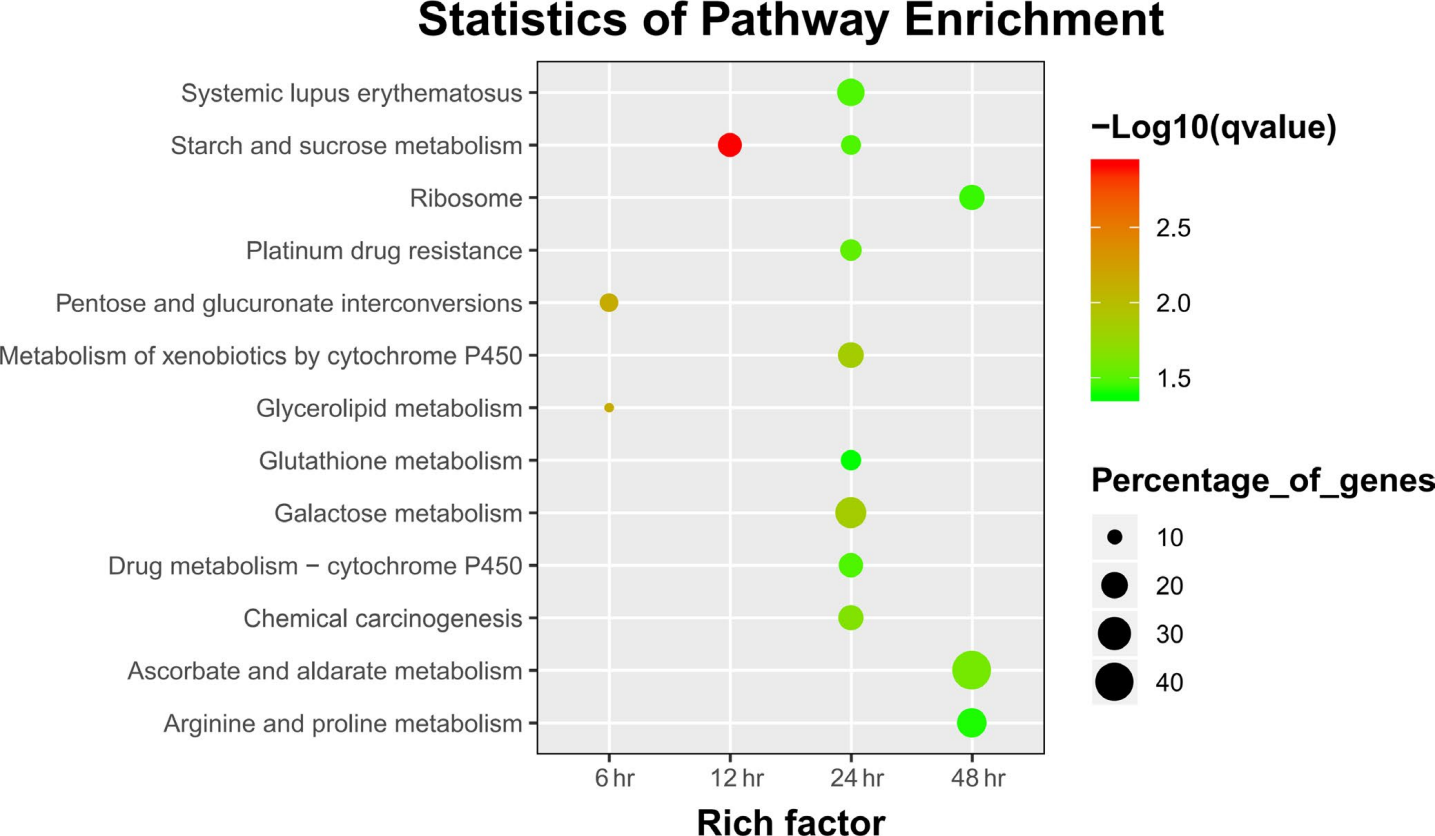
KEGG term enrichments of fungal DEGs during the algicidal process

After the comparison of candidate genes with Nr from NCBI, GO, and KEGG databases, DEGs were used to obtain enriched terms by Fisher's exact test (*p* < .05). The GO terms of DEGs were enriched in the extracellular region, cell wall, signal recognition particle, proteasome core complex, prefold in complex, ribosome, and other cellular components categories (Figure 4). Similarly, DEGs were found to be enriched on transport and catabolic processes in the biological process category, particularly cellulose catabolism and carbohydrate transport (Figure 5). Further, DEGs were enriched on decomposition and transporter activities in the molecular function category that included the activities of triglyceride lipase, serine‐type peptidase, manganese peroxidase, carboxypeptidase, cellulose 1,4‐β‐cellobiosidase, β‐glucosidase, aspartic‐type endopeptidase, α‐amylase, glycolipid transporter, amino acid transmembrane transporter, and other (Figure 6). The KEGG analysis showed that DEGs were enriched on glycerolipid metabolism, starch and sucrose metabolism, metabolism of xenobiotics by cytochrome P450, galactose metabolism, and ascorbate and aldarate metabolism in different stages of the algicidal process (Figure 7).

### 3.5 Composition and expression of CAZyme genes of *B. adusta* T1 and its comparison with that of *T. versicolor* F21a

A total of401 CAZyme genes were identified in the genome of *B. adusta* by hmmscan against the dbCAN database (Table 1). The lignocellulose‐active genes can be divided into 77 CAZyme modules (Table 1). Most of the genes belonged to Glycoside Hydrolases (GH) family and Auxiliary Activities (AA) family. About 312 CAZyme genes were identified in the genome of *T. versicolor* F21a (Dai et al., 2018). The number of CAZyme genes in *B. adusta* T1 genome (401 CAZyme genes) was higher than that of *T. versicolor* F21a (312 CAZyme genes). Seventy CAZyme modules were detected in *B. adusta* T1, compared to 43 CAZyme modules in *T. versicolor* F21a in the previous study (Dai et al., 2018). However, the algicidal effects of *T. versicolor* F21a were slightly more efficient than that of *B. adusta* T1 (Han et al., 2011).

**TABLE 1:** The number of decomposition enzymes detected by RNA‐Seq

Enzyme classes	CAZyme module	No. of decomposition enzymes in the genome	No. of decomposition enzymes detected by RNA‐Seq	No. of decomposition enzymes in DEGs by RNA‐Seq
Auxiliary activities	AA1	1	1	
	AA2	21	19	10
	AA3	38	30	12
	AA4	1		
	AA5	7	8	6
	AA6	5	4	3
	AA7	10	6	3
	AA8	2	2	
	AA9	27	20	7
Carbohydrate esterases	CE1	18	11	3
	CE10	42	31	6
	CE12	3	2	
	CE14	1	1	
	CE15	2	2	
	CE16	14	6	3
	CE2	1	1	
	CE3	1	1	
	CE4	5	3	3
	CE8	2	2	1
	CE9	1		
	GH1	2	2	1
Glycoside hydrolases	GH10	4	5	4
	GH105	3	3	1
	GH109	8	8	5
	GH115	2	2	1
	GH12	2	1	
	GH125	1	1	
	GH127	1	1	
	GH128	5	3	2
	GH13	9	9	6
	GH131	3		
	GH15	2	2	
	GH16	19	17	5
	GH17	1	1	
	GH18	13	10	3
	GH2	3	2	2
	GH20	4	2	
	GH23	1		
	GH24	1	1	
	GH25	1	1	
	GH27	3	3	1
	GH28	6	4	
	GH3	8	8	4
	GH30	1	1	1
	GH31	4	5	3
	GH35	4	4	
	GH37	2	1	1
	GH38	1		
	GH43	6	6	4
	GH47	6	3	
	GH5	20	16	8
	GH51	2	2	1
	GH53	1	1	
	GH55	3	3	1
	GH6	1	1	1
	GH63	2	1	
	GH7	5	4	1
	GH71	3	3	1
	GH72	1	1	
	GH74	3	3	
	GH76	2	1	
	GH78	2	2	
	GH79	7	9	6
	GH85	1	1	
	GH88	1	1	
	GH89	1	1	
	GH9	1	1	
	GH92	3	3	1
	GH95	1	1	
	GH99	1		
Polysaccharide lyases	PL1	1	1	
	PL12	1	1	
	PL14	5	6	5
	PL3	2	2	
	PL4	1		
	PL5	1	2	2
	PL8	1	1	
	Total	401	324	128

**FIGURE 8:**
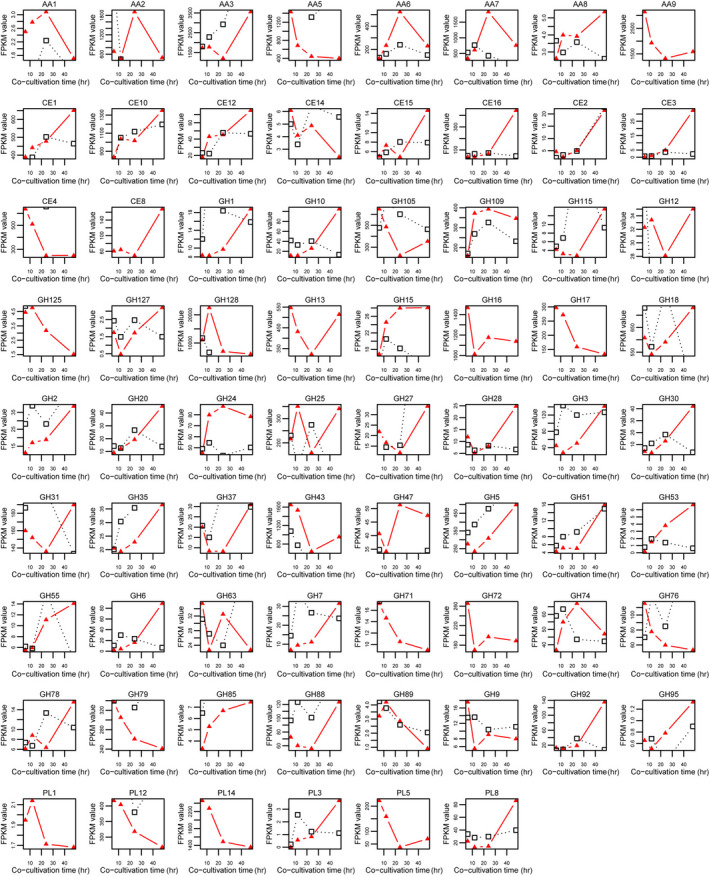
Total expression levels of each CAZyme module during the algicidal process

The identified 128 differentially expressed CAZyme genes in *B. adusta* T1 were found to belong to 37 modules (Table 1). The genes within the same module exhibited diverse expression profiles during the algicidal process of *B. adusta* T1 (Figure 8). It was observed that module GH128, AA7, AA6, and GH109 had the highest accumulated expression during the algicidal process. The sublocation analysis showed that ~ 61% (245/401) of lignocellulose‐active proteins contained secretory pathway signal peptides that can be secreted outside of fungal mycelia (Table A3). Genes within GH128 that encoded endo‐1,3‐β‐glucanase (EC3.2.1.39) could decompose xyloglucans and β‐1,3‐glucans into xylose and glucose, respectively. The enzymes of GH128, AA7, AA6, and GH109 were less efficient in cyanobacterial cell disruption. It is noteworthy that the accumulated expression of Polysaccharide lyases genes, particularly the PL8 module was highly up‐regulated during the later stage of the algicidal process of *B. adusta* T1, which was much delayed when compared to *T. versicolor* F21a (Dai et al., 2018).

### 3.6 Expression of other decomposition genes in *B. adusta* T1 and their comparison with that of *T. versicolor* F21a

**FIGURE 9:**
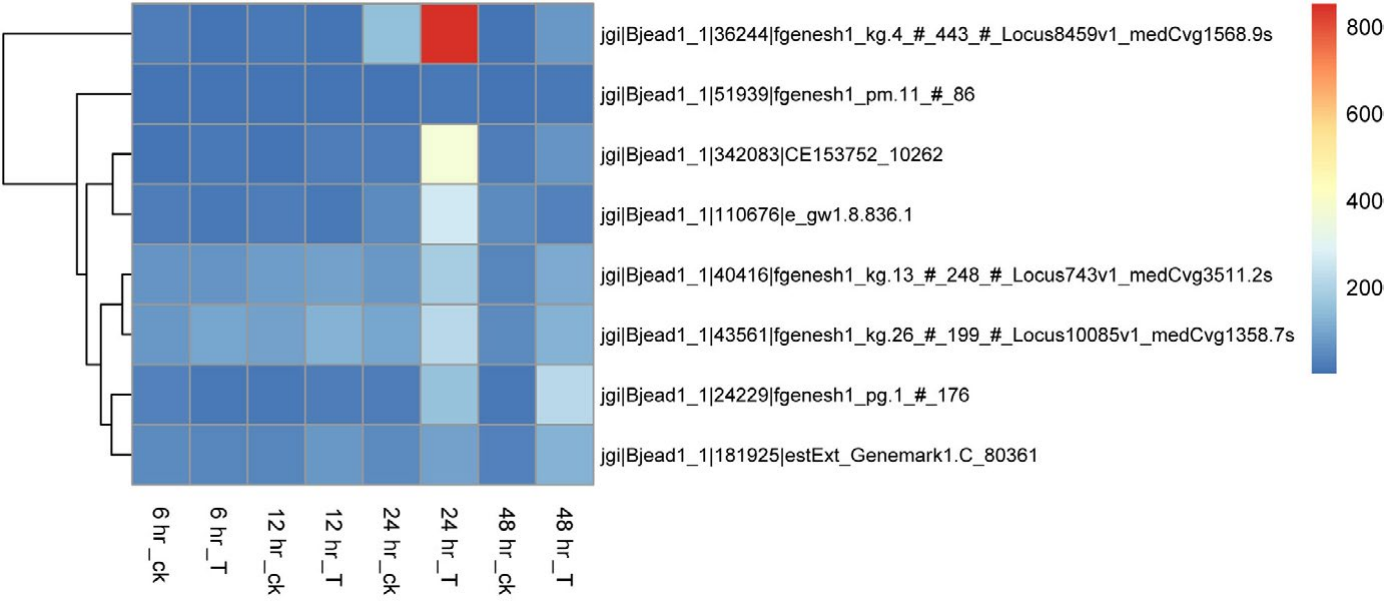
Time‐course change of protease genes expression level of T1 cocultivation with cyanobacteria. Note: 6h_ck, control sample at 6h; 6h_T, treatment sample at 6 hr; 12h_ck, control sample at 12 hr; 12h_T, treatment sample at 12 hr; 24h_ck, control sample at 24 hr; 24h_T, treatment sample at 24 hr; 48h_ck, control sample at 48 hr; 48h_T, treatment sample at 48 hr

Only a few serine‐type peptidase, carboxypeptidase, and aspartic‐type endopeptidase, with strong ability in cyanobacterial cells disruption, were enriched in the DEGs list during the early stage of the algicidal process (6 hr) (Figure 6). However, no strong decomposition enzyme was enriched during the later stage of the algicidal process until 24 hr (Figure 6). During the later stage (24 hr), proteins with aspartic‐type endopeptidase activity and manganese peroxidase activity were the main decomposition enzymes (Figure 6). Various types of decomposition enzymes, such as threonine‐type endopeptidase and serine‐type endopeptidase, were induced after 48 hr of cocultivation. In this study, proteases with Protein ID jgi|Bjead1_1|36244|fgenesh1_kg.4_#_443_#_Locus8459v1_medCvg1568.9s and jgi|Bjead1_1|342083|CE153752_10262, and jgi|Bjead1_1|110676|e_gw1.8.836.1 were observed to be the main degradation genes that might be involved in cyanobacterial cells disruption (Figure 9). Thus, these proteases can play significant roles in the algicidal process. The decomposition genes showed delayed expression compared with that of *T. versicolor* F21a.

## 4 DISCUSSION

Although several fungi showed a strong algicidal activity (Han et al., 2011), the underlying molecular mechanisms for algicidal capacities are largely less investigated. Interestingly, a few fungi from the Polyporales order of Basidiomycota exhibited a strong algicidal activity (Han et al., 2011). Comparative genome analyses found that the genomes of white rot fungi contain more genes encoding plant cell wall degrading enzymes than that of brown rot and mycorrhizal fungi (Kohler et al., 2015; Tisserant et al., 2013). White rot fungi including the order Polyporales can degrade lignin as well as cellulose (Kohler et al., 2015). In the present study, we observed that the number of CAZyme genes and expressed CAZyme genes of *B. adusta* T1 was great than that of *T. versicolor* F21a. However, the algicidal effects of *B. adusta* T1 were slightly less efficient than that of *T. versicolor* F21a (Han et al., 2011). More genome sequences of fungi with diverse algicidal abilities are available now, and we also compared the number of CAZyme genes in the genome of different algicidal fungi. No direct correlation was found between algicidal efficiency and several CAZyme genes (Data not shown). A similar result was observed in the study of Pilgaard et al., 2019. This suggested that the high efficiencies of algicidal fungi are not attributed to the number of genes encoding CAZyme in the fungal genome. High lignocellulose degradation ability of white rot fungi, in comparison with that of brown rot fungi and mycorrhizal fungi, can be attributed to the number of genes encoding plant cell wall degrading enzymes in fungal genomes as a result of long term natural selection (Kohler et al., 2015). The numbers of CAZyme genes were not directly correlated with algicidal abilities, which might be due to the fact that most algicidal fungi were isolated from terrestrial environments and lacked evolution selection pressure in the water system (Han et al., 2011).

Direct contact between fungal mycelia and cyanobacterial cells was required for eliminating cyanobacterial cells by fungi (Han et al., 2011; Jia et al., 2010). Previous studies showed that a few decomposition enzymes might play important roles in eliminating cyanobacterial cells by *T. versicolor* F21a. In particular, cellulase, β‐glucanase, and protease were supposed to efficiently disrupt cyanobacterial cells by *T. versicolor* F21a (Dai et al., 2018; Gao et al., 2017). In the present study, a large number of decomposition enzymes belonging to 37 modules were observed during the algicidal process of *B. adusta* T1. Among them, GH128, AA7, AA6, and GH109 were the highest accumulated expression module. However, the enzymes of GH128, AA7, AA6, and GH109 were not able to efficiently disrupt the macromolecules (Ekstrom, Taujale, McGinn, & Yin, 2014; Yin et al., 2012), such as cellulose in the cell wall of cyanobacterial cells. This suggested that lignocellulose‐active proteins of *B. adusta* T1 might not be the key enzymes for the breakdown of cyanobacterial cells.

Previous studies showed that chondroitin ABC lyase (EC4.2.2.1) of PL8 and alginate lyase (EC4.2.2.3) of PL14 were able to decompose peptidoglycan and alginate (Lombard, Golaconda Ramulu, Drula, Coutinho, & Henrissat, 2014), and the expression level was also significantly up‐regulated during the algicidal process of *T. versicolor* F21a (Dai et al., 2018; Gao et al., 2017). Chondroitin AC lyase (chondroitin sulfate) and alginate lyase were unique to a known saprophytic marine fungus *Paradendryphiella salina* in the breakdown of dried brown algae in the medium compared with its terrestrial counterparts (Pilgaard et al., 2019). Recombinant expression of Chondroitin AC lyase of the marine fungus *P.salina* reveals that alginate lyase can degrade several types of brown algae polysaccharides (Pilgaard et al., 2019). A putative PL8 of *P.salina* with a similar sequence should also decompose brown macroalgae (Pilgaard et al., 2019). Proteomic analysis of the secretome of *P. salina* grown on three species of brown algae and under carbon limitation implied that the basic CAZyme repertoire of saprobic fungi belongs to ascomycetes, with the addition of PL7 alginate lyases, provide *P. salina* with sufficient enzymatic capabilities to degrade several types of brown algae polysaccharides (Pilgaard et al., 2019). In the present study, the total expression level of PL14 was down‐regulated during the algicidal process of *B. adusta* T1, while no gene, belonging to PL7, was detected in the genome of *B. adusta*. The accumulated expression level of PL8 was highly up‐regulated in the later stage of the algicidal process of *B. adusta* T1, which was much delayed when compared with *T. versicolor* F21a (Dai et al., 2018). All the evidence indicated that enzymes of PL8 with strong peptidoglycan and alginate decomposition abilities might be a vital genetic factor for the determination of the algicidal ability of *T. versicolor* F21a as well as *B. adusta* T1.

Analysis of the enriched GO terms and KEGG pathways showed that several types of peptidases were enriched during the algicidal process of *B. adusta* T1. In particular, proteases (protein ID jgi|Bjead1_1|36244|fgenesh1_kg.4_#_443_#_Locus8459v1_medCvg1568.9s, jgi|Bjead1_1|342083|CE153752_10262, and jgi|Bjead1_1|110676|e_gw1.8.836.1) were highly up‐regulated during the later stages of cocultivation. Proteomic analysis of *P. salina* also implied that the PL7 and PL8 enzymes, abundantly secreted together with enzymes of *P.salina,* were necessary for degradation of laminarin, cellulose, lipids, and peptides of brown algae (Pilgaard et al., 2019). Different types of peptides were detected in *P. salina* grown on three species of brown algae (Pilgaard et al., 2019). Additionally, several fungal proteins belonging to peptidase were also up‐regulated during the algicidal process of *T. versicolor* F21a (Gao et al., 2017). Besides, four homologous decomposition enzymes of other species with endo‐glycosidase and endopeptidase activities were selected to investigate their effects on cyanobacterial cells, and one type of protease was found to effectively disrupt cyanobacterial cells (Dai et al., 2018). Comparison of the gene expression during the algicidal process of *B. adusta* T1 and *T. versicolor* F21a demonstrated that majority of decomposition genes with endopeptidase and endo‐glycosidase activities in *B. adusta* T1 were expressed in the later stage of cocultivation, while the similar genes in *T. versicolor* F21a were induced in the early stage (Dai et al., 2018). Thus, protease together with enzymes of PL8 might play a key role in the elimination of cyanobacterial cells both by *B. adusta* T1 and *T. versicolor* F21a. The expression of enzymes of PL8 and peptidases in *B. adusta* T1 was little delayed compared with that of *T. versicolor* F21a, which should be the reason why the algicidal efficiency of *T. versicolor* F21a is better than that of *B. adusta* T1.

The production of microcystins (MC) by cyanobacterial blooms often severely threatens human and ecosystems health (Li, Li, & Li, 2017). Biodegradation is an efficient and sustainable biological strategy for MC removal (Li et al., 2017). A large number of bacteria and several fungi were reported with MC removal or degrading capabilities (Dziga, Wasylewski, Wladyka, Nybom, & Meriluoto, 2013; Jia, Du, Song, Zhao, & Tian, 2012; Li et al., 2017; Mohamed, Hashem, & Alamri, 2014; Qin et al., 2019). Four *mlr* genes (i.e., *mlrC*, *A*, *D,* and *B*) located sequentially in a gene cluster in the genome of *Sphingomonas* sp. ACM‐3962 strain were identified for MC biodegradation (Bourne et al., 1996; Bourne, Riddles, Jones, Smith, & Blakeley, 2001). The enzymatic pathway involves at least three intracellular enzymes and two intermediate products (Li et al., 2017). Heterologous expression of the *mlrA* gene originated from *Novosphingobium* sp. THN1 showed that the recombinant MlrA hydrolyzed microcystin‐RR into a linear intermediate product by cleaving the peptide bond between Adda and arginine residue, which is also the first step involved in MC degradation pathway (Wang et al., 2017). Site‐directed mutants of MlrA suggested that MlrA is likely not a metalloprotease but a glutamate protease belonging to type II CAAX prenyl endopeptidases (Xu et al., 2019). A few fungi, for example, *T. abietinum* 1302BG, *T.citrinoviride*, and *Mucor hiemalis* were reported with MC removal or degrading capability (Esterhuizen‐Londt, Hertel, & Pflugmacher, 2017; Jia et al., 2012; Mohamed et al., 2014; Stephan, 2015); however, the enzymatic pathway was poorly understood compared with that of bacteria. In our study, many genes with endopeptidase activities were enriched during the algicidal process, and a gene encoding aflatoxin‐detoxifizyme with peptidase activity (Protein ID: jgi|Bjead1_1|37717|fgenesh1_kg.7_#_39_#_Locus4370v1_medCvg2101.1s) was up‐regulated during the algicidal process of *B. adusta* T1. Further mining the gene expression during the algicidal process of *T. versicolor* F21a identified a homolog gene (Protein ID: jgi|Trave1|56726|estExt_fgenesh1_pm.C_3_t10209) that was slightly up‐regulated in the later stage. In consideration bacterial MlrA encoding a protease, fungal aflatoxin‐detoxifizyme could be a possible candidate enzyme involving in MC degradation. In order to investigate the mechanism for MC degradation in fungi, there is more work need to be done.

## 5 CONCLUSIONS

In this study, the algicidal process of *B. adusta* T1 was investigated by a time‐serial transcriptomic analysis, and the results were compared with these from *T. versicolor* F21a, reported in our previous study. The identified DEGs were enriched in endopeptidase activity, cellulose catabolic process, and transmembrane transporter activity. Endopeptidases together with enzymes of PL8 might play a key role in the elimination of cyanobacterial cells by both algicidal fungi, *B. adusta* T1 and *T. versicolor* F21a.

## CONFLICTS OF INTEREST

None declared.

## AUTHOR CONTRIBUTION

**Guomin Han:** Conceptualization (equal); Software (lead); Writing‐original draft (equal). **Hui Ma:** Investigation (equal). **Shenrong Ren:** Investigation (supporting). **Xueyan Gao:** Investigation (supporting). **Xiaolong He:** Investigation (supporting). **Suwen Zhu:** Resources (equal); Validation (supporting). **Ruining Deng:** Validation (supporting). **Shihua Zhang:** Conceptualization (equal); Writing‐review & editing (equal). 

## ETHICS STATEMENT

None required.

## Data Availability

The raw paired‐end sequences from the *Bjerkandera adusta* isolate T1 are available at https://www.ncbi.nlm.nih.gov/bioproject/PRJNA543936. The genome annotations of *Bjerkandera adusta* and *Trametes versicolor* can be found at the JGI MycoCosm: https://mycocosm.jgi.doe.gov/Bjead1_1/Bjead1_1.home.html and https://mycocosm.jgi.doe.gov/Trave1/Trave1.home.html, respectively.

## APPENDIX 1

**TABLE A1:** Primers used in this study

Protein ID	Annotation	Primer
jgi|Bjead1_1|459664|MIX10988_17319_14	Radical oxidase	GTCGAAGCGGGTGGTCTTAA
		CCTCTCCTCGTTGCCGTTT
jgi|Bjead1_1|34143|fgenesh1_kg.1_#_945_#_Locus732v1_medCvg1115.6s	Esterase family 1 protein	CCTCCCTGCAAACATCTCACA
		GGAGACGTGTCGGGAAAGAG
jgi|Bjead1_1|172436|gm1.8875_g	Hydrolase family 5 protein	TACGAGGGCGACGATTGG
		CTCACCGGACACGTAAACCA
jgi|Bjead1_1|35099|fgenesh1_kg.2_#_711_#_Locus118v3_medCvg9284.2s	Hydrolase family 5 protein	CTCGTTGACCCGCACAACTT
		GGGAATATCGTGAGGCTCGTT
jgi|Bjead1_1|355947|CE167616_517	Hydrolase family 128 protein	AGCGCGGTGTGTCATACAAC
		TGTGTCCGGCATCGGTATT
jgi|Bjead1_1|38229|fgenesh1_kg.7_#_551_#_Locus8080v1_medCvg1578.8s	Hydrolase family 13 protein	CACGCCCGACTATTCGAAGT
		GTCGGGTTTTCCGTGTCAAG

**TABLE A2:** Statistics of RNA‐Seq reads mapping results

Sample	Raw reads	Number of input reads	Cleaned length	Uniquely mapped reads number	Uniquely mapped reads (%)
6h_ck1	3,713,910	3,531,468	129.69	2,491,981	70.57
6h_ck2	3,618,291	3,416,852	129.055	2,205,290	64.54
6h_T1	3,644,390	3,577,152	128.745	2,802,466	78.34
6h_T2	4,379,858	4,280,639	128.835	3,282,569	76.68
12h_ck1	3,832,620	3,691,873	129.505	2,706,111	73.30
12h_ck2	3,806,801	3,651,869	129.09	2,603,461	71.29
12h_T1	3,493,777	3,325,461	125.865	2,458,201	73.92
12h_T2	4,020,571	3,899,676	128.9	2,967,516	76.10
24h_ck1	3,609,635	3,388,118	128.875	2,326,955	68.68
24h_ck2	3,684,973	3,497,767	129.655	2,466,212	70.51
24h_T1	4,831,627	4,684,474	128.62	3,554,342	75.87
24h_T2	4,567,295	4,436,833	128.9	3,395,508	76.53
48h_ck1	3,638,776	3,456,573	129.255	2,500,095	72.33
48h_ck2	3,594,592	3,405,731	128.51	2,473,909	72.64
48h_T1	4,499,718	4,347,471	128.275	3,264,957	75.10
48h_T2	4,500,181	4,353,762	128.58	3,277,084	75.27

Note

The number of reads were expressed in pairs.

**TABLE A3:** Sublocation of CAZyme proteins of *B. adusta*

Protein ID	Len	mTP	SP	Other	Loc	RC
39948	1,041	0.095	0.093	0.844	_	2
170203	646	0.083	0.105	0.872	_	2
229483	319	0.049	0.917	0.053	S	1
113359	295	0.045	0.95	0.032	S	1
40021	320	0.081	0.908	0.028	S	1
40040	465	0.053	0.942	0.029	S	1
230253	1,024	0.014	0.966	0.07	S	1
230354	1,005	0.342	0.705	0.024	S	4
183239	385	0.671	0.027	0.355	M	4
62585	305	0.147	0.104	0.761	_	2
113961	604	0.534	0.055	0.439	M	5
452849	310	0.077	0.037	0.944	_	1
170455	322	0.091	0.068	0.894	_	1
237378	316	0.042	0.949	0.058	S	1
40461	244	0.054	0.954	0.043	S	1
183509	612	0.558	0.024	0.588	_	5
240122	301	0.092	0.873	0.031	S	2
40615	587	0.037	0.159	0.919	_	2
241975	605	0.068	0.073	0.901	_	1
52811	537	0.063	0.913	0.03	S	1
40743	377	0.103	0.892	0.017	S	2
170929	704	0.088	0.048	0.937	_	1
170934	551	0.063	0.897	0.086	S	1
244200	674	0.027	0.93	0.065	S	1
244246	669	0.018	0.971	0.054	S	1
62986	499	0.058	0.906	0.041	S	1
71431	617	0.491	0.658	0.014	S	5
245049	604	0.442	0.655	0.01	S	4
40812	611	0.087	0.044	0.906	_	1
245297	598	0.061	0.81	0.11	S	2
171002	606	0.196	0.68	0.028	S	3
84503	373	0.101	0.05	0.922	_	1
171059	593	0.14	0.872	0.019	S	2
156054	596	0.044	0.887	0.074	S	1
114954	574	0.084	0.115	0.897	_	2
40886	614	0.079	0.052	0.904	_	1
136631	614	0.123	0.045	0.86	_	2
114902	593	0.423	0.556	0.029	S	5
52983	613	0.052	0.044	0.95	_	1
52991	597	0.044	0.914	0.052	S	1
183896	599	0.014	0.93	0.089	S	1
53087	1,011	0.036	0.969	0.05	S	1
41108	696	0.159	0.081	0.841	_	2
41113	573	0.093	0.207	0.634	_	3
171368	478	0.069	0.079	0.9	_	1
41241	396	0.274	0.84	0.018	S	3
454703	402	0.63	0.021	0.452	M	5
41251	337	0.075	0.736	0.21	S	3
41305	371	0.11	0.094	0.754	_	2
41306	538	0.094	0.099	0.816	_	2
256509	423	0.468	0.891	0.004	S	3
184224	600	0.103	0.101	0.838	_	2
41490	303	0.052	0.147	0.93	_	2
260893	199	0.086	0.089	0.914	_	1
184394	582	0.803	0.053	0.115	M	2
157149	768	0.029	0.956	0.036	S	1
41596	266	0.069	0.929	0.046	S	1
171769	774	0.021	0.96	0.057	S	1
261859	808	0.085	0.06	0.922	_	1
116111	281	0.069	0.143	0.873	_	2
263236	400	0.054	0.958	0.066	S	1
29758	400	0.043	0.995	0.011	S	1
263252	398	0.053	0.98	0.022	S	1
41686	427	0.307	0.369	0.337	S	5
41708	649	0.18	0.862	0.014	S	2
41754	647	0.053	0.182	0.858	_	2
41763	404	0.094	0.768	0.13	S	2
53682	693	0.255	0.759	0.029	S	3
41854	517	0.021	0.968	0.058	S	1
41863	491	0.11	0.913	0.016	S	1
41869	336	0.222	0.908	0.016	S	2
41896	447	0.713	0.025	0.412	M	4
138203	774	0.05	0.127	0.857	_	2
184697	372	0.085	0.874	0.045	S	2
116816	362	0.078	0.863	0.061	S	2
172102	377	0.068	0.887	0.051	S	1
41961	329	0.044	0.92	0.064	S	1
456042	328	0.038	0.942	0.045	S	1
268970	386	0.025	0.953	0.056	S	1
29957	283	0.019	0.958	0.067	S	1
116945	203	0.143	0.062	0.889	_	2
157771	401	0.037	0.944	0.045	S	1
157775	304	0.039	0.933	0.056	S	1
63838	343	0.015	0.974	0.051	S	1
41982	373	0.156	0.807	0.026	S	2
172152	362	0.046	0.927	0.051	S	1
269481	367	0.124	0.787	0.049	S	2
269524	373	0.13	0.823	0.028	S	2
41997	618	0.09	0.958	0.02	S	1
172246	372	0.117	0.854	0.028	S	2
157924	374	0.155	0.857	0.027	S	2
117149	396	0.013	0.496	0.863	_	4
184935	309	0.034	0.944	0.061	S	1
138704	475	0.096	0.071	0.887	_	2
172436	486	0.083	0.049	0.928	_	1
42291	347	0.056	0.938	0.021	S	1
81341	141	0.059	0.274	0.852	_	3
158334	414	0.237	0.054	0.674	_	3
42434	421	0.05	0.914	0.051	S	1
54172	363	0.017	0.977	0.039	S	1
185179	452	0.09	0.805	0.059	S	2
117666	259	0.056	0.913	0.05	S	1
42534	327	0.084	0.883	0.031	S	2
42539	270	0.19	0.044	0.855	_	2
296151	848	0.178	0.112	0.76	_	3
185311	397	0.037	0.706	0.59	S	5
42617	504	0.024	0.239	0.872	_	2
42631	975	0.141	0.86	0.023	S	2
117772	330	0.454	0.018	0.718	_	4
54399	313	0.027	0.948	0.043	S	1
172925	348	0.068	0.979	0.031	S	1
172926	355	0.031	0.968	0.045	S	1
158817	338	0.037	0.936	0.087	S	1
158842	1,102	0.131	0.059	0.88	_	2
185485	287	0.099	0.149	0.826	_	2
118319	648	0.118	0.832	0.057	S	2
139564	387	0.947	0.041	0.047	M	1
42889	285	0.055	0.192	0.895	_	2
302552	344	0.191	0.044	0.811	_	2
305292	253	0.084	0.889	0.033	S	1
43095	366	0.129	0.816	0.031	S	2
306404	366	0.113	0.849	0.032	S	2
43114	348	0.099	0.818	0.057	S	2
306863	366	0.041	0.901	0.056	S	1
118718	363	0.052	0.884	0.049	S	1
119037	314	0.05	0.91	0.069	S	1
43329	364	0.143	0.843	0.03	S	2
173495	364	0.337	0.782	0.013	S	3
311850	437	0.082	0.904	0.031	S	1
54893	416	0.149	0.835	0.047	S	2
185921	568	0.19	0.847	0.046	S	2
459664	777	0.052	0.777	0.257	S	3
43446	386	0.099	0.882	0.061	S	2
313682	859	0.047	0.95	0.031	S	1
173673	260	0.015	0.968	0.041	S	1
119350	399	0.033	0.941	0.062	S	1
119522	575	0.905	0.042	0.13	M	2
119593	1,000	0.12	0.864	0.027	S	2
43812	1,034	0.027	0.834	0.427	S	3
43892	615	0.019	0.971	0.047	S	1
323280	369	0.239	0.763	0.019	S	3
43929	258	0.026	0.965	0.084	S	1
186344	320	0.122	0.849	0.039	S	2
120002	362	0.08	0.87	0.048	S	2
55334	249	0.034	0.907	0.075	S	1
43966	742	0.024	0.946	0.05	S	1
324420	819	0.032	0.934	0.049	S	1
186388	434	0.136	0.879	0.039	S	2
44047	495	0.025	0.966	0.045	S	1
44072	557	0.412	0.595	0.018	S	5
326659	470	0.38	0.617	0.062	S	4
141290	460	0.062	0.147	0.889	_	2
120399	298	0.117	0.362	0.535	_	5
344867	663	0.092	0.927	0.021	S	1
141539	663	0.101	0.904	0.022	S	1
345914	804	0.294	0.842	0.009	S	3
44370	571	0.236	0.777	0.041	S	3
44376	532	0.037	0.964	0.029	S	1
44391	385	0.022	0.964	0.048	S	1
141648	466	0.369	0.686	0.046	S	4
55696	466	0.59	0.689	0.03	S	5
462628	730	0.223	0.087	0.657	_	3
120968	1,020	0.018	0.966	0.057	S	1
161363	452	0.052	0.912	0.047	S	1
174734	531	0.054	0.857	0.206	S	2
161500	326	0.05	0.927	0.032	S	1
353490	284	0.807	0.044	0.166	M	2
353489	254	0.422	0.051	0.632	_	4
44803	1,134	0.105	0.028	0.928	_	1
355947	264	0.021	0.946	0.08	S	1
73811	287	0.489	0.745	0.016	S	4
121664	369	0.079	0.874	0.033	S	2
31936	332	0.211	0.783	0.03	S	3
73869	357	0.123	0.12	0.842	_	2
463744	931	0.029	0.972	0.025	S	1
45029	958	0.016	0.969	0.057	S	1
187270	615	0.044	0.957	0.029	S	1
56225	960	0.166	0.202	0.582	_	4
32051	406	0.186	0.073	0.732	_	3
361367	713	0.08	0.063	0.949	_	1
73972	481	0.159	0.698	0.092	S	3
175283	521	0.208	0.945	0.004	S	2
45135	778	0.166	0.895	0.011	S	2
121936	537	0.043	0.918	0.053	S	1
45153	588	0.043	0.845	0.111	S	2
122105	500	0.097	0.128	0.838	_	2
56307	208	0.323	0.115	0.412	_	5
45281	340	0.341	0.59	0.036	S	4
45314	601	0.082	0.12	0.844	_	2
465711	611	0.071	0.279	0.729	_	3
175513	700	0.052	0.367	0.644	_	4
143000	604	0.101	0.128	0.746	_	2
56449	403	0.04	0.96	0.034	S	1
175536	379	0.127	0.872	0.031	S	2
74164	587	0.144	0.145	0.649	_	3
162505	587	0.081	0.165	0.796	_	2
56499	798	0.368	0.808	0.011	S	3
56525	330	0.449	0.653	0.023	S	4
45516	850	0.067	0.891	0.081	S	1
162602	215	0.09	0.086	0.866	_	2
384658	698	0.142	0.879	0.033	S	2
45570	1,468	0.018	0.965	0.042	S	1
187728	605	0.116	0.157	0.718	_	3
45647	404	0.1	0.692	0.179	S	3
66377	626	0.158	0.64	0.062	S	3
66400	890	0.144	0.023	0.921	_	2
122937	361	0.08	0.919	0.03	S	1
66493	204	0.046	0.304	0.687	_	4
143585	374	0.045	0.094	0.947	_	1
123323	650	0.114	0.295	0.801	_	3
45905	313	0.127	0.925	0.028	S	2
403554	339	0.262	0.075	0.74	_	3
56859	320	0.081	0.863	0.059	S	2
176420	458	0.019	0.972	0.037	S	1
46260	847	0.035	0.189	0.908	_	2
188241	862	0.038	0.161	0.94	_	2
33215	801	0.067	0.777	0.182	S	3
101267	513	0.022	0.946	0.089	S	1
33263	449	0.021	0.9	0.099	S	1
102985	338	0.067	0.974	0.027	S	1
448899	540	0.413	0.042	0.616	_	4
177450	748	0.043	0.939	0.031	S	1
196330	544	0.05	0.961	0.019	S	1
33636	717	0.168	0.175	0.701	_	3
125362	239	0.358	0.042	0.545	_	5
100935	396	0.091	0.352	0.583	_	4
164180	836	0.303	0.845	0.012	S	3
102479	750	0.243	0.06	0.721	_	3
199563	388	0.678	0.27	0.052	M	3
145317	557	0.044	0.799	0.201	S	3
95645	99	0.09	0.193	0.743	_	3
33906	312	0.19	0.087	0.732	_	3
201958	607	0.826	0.018	0.328	M	3
33959	594	0.708	0.032	0.35	M	4
33963	340	0.025	0.921	0.133	S	2
203296	422	0.12	0.923	0.024	S	1
34143	292	0.061	0.079	0.934	_	1
47402	390	0.028	0.942	0.071	S	1
164550	682	0.506	0.434	0.072	M	5
126363	785	0.226	0.14	0.716	_	3
34175	506	0.937	0.026	0.099	M	1
126440	419	0.058	0.137	0.91	_	2
207338	523	0.042	0.926	0.053	S	1
34226	479	0.071	0.086	0.922	_	1
207890	208	0.042	0.926	0.053	S	1
24753	992	0.257	0.036	0.723	_	3
101242	366	0.165	0.049	0.851	_	2
209426	255	0.073	0.226	0.761	_	3
47558	367	0.035	0.916	0.062	S	1
164740	337	0.048	0.798	0.118	S	2
47647	744	0.034	0.946	0.075	S	1
103882	413	0.153	0.052	0.854	_	2
24940	781	0.104	0.077	0.907	_	1
34577	474	0.223	0.03	0.844	_	2
24950	374	0.049	0.978	0.015	S	1
275330	650	0.465	0.629	0.023	S	5
34622	607	0.147	0.12	0.719	_	3
34651	526	0.127	0.039	0.875	_	2
34705	653	0.061	0.067	0.905	_	1
165147	577	0.063	0.086	0.896	_	1
34805	391	0.914	0.035	0.118	M	2
280856	545	0.204	0.073	0.755	_	3
104675	505	0.097	0.869	0.039	S	2
282706	466	0.089	0.952	0.038	S	1
34945	466	0.261	0.722	0.022	S	3
35099	397	0.034	0.957	0.044	S	1
35123	603	0.73	0.055	0.209	M	3
35255	335	0.09	0.869	0.039	S	2
128174	279	0.124	0.95	0.01	S	1
331356	251	0.066	0.79	0.195	S	3
35327	233	0.037	0.887	0.112	S	2
35330	235	0.04	0.881	0.096	S	2
104983	435	0.365	0.084	0.512	_	5
165879	280	0.063	0.095	0.909	_	1
165993	546	0.072	0.707	0.106	S	2
338580	678	0.048	0.947	0.019	S	1
35711	528	0.089	0.177	0.79	_	2
105145	362	0.361	0.72	0.031	S	4
48723	546	0.07	0.964	0.021	S	1
35742	516	0.053	0.457	0.796	_	4
340063	377	0.193	0.176	0.709	_	3
105469	346	0.204	0.051	0.772	_	3
105723	203	0.124	0.097	0.841	_	2
48765	461	0.582	0.076	0.307	M	4
105560	432	0.053	0.904	0.049	S	1
68408	564	0.03	0.956	0.04	S	1
25772	321	0.025	0.966	0.043	S	1
106998	404	0.052	0.172	0.887	_	2
35876	325	0.046	0.921	0.085	S	1
35880	488	0.054	0.182	0.909	_	2
106046	321	0.103	0.937	0.018	S	1
106351	275	0.118	0.059	0.875	_	2
59360	863	0.079	0.83	0.127	S	2
25843	321	0.303	0.882	0.013	S	3
166233	323	0.181	0.857	0.033	S	2
35905	325	0.061	0.937	0.04	S	1
129150	325	0.167	0.909	0.032	S	2
364963	340	0.055	0.899	0.036	S	1
365447	826	0.077	0.893	0.039	S	1
365822	509	0.036	0.921	0.071	S	1
106230	542	0.345	0.068	0.679	_	4
464718	327	0.1	0.856	0.041	S	2
49096	474	0.051	0.961	0.044	S	1
129655	389	0.274	0.187	0.411	_	5
49205	779	0.028	0.954	0.046	S	1
166629	301	0.351	0.134	0.447	_	5
180053	292	0.4	0.1	0.395	M	5
107081	1,018	0.021	0.962	0.059	S	1
106859	396	0.04	0.484	0.631	_	5
387673	192	0.127	0.115	0.864	_	2
107229	219	0.14	0.06	0.876	_	2
107188	503	0.053	0.868	0.154	S	2
108447	882	0.021	0.947	0.069	S	1
36572	867	0.082	0.087	0.879	_	2
389256	583	0.057	0.958	0.017	S	1
49473	238	0.019	0.964	0.071	S	1
180279	701	0.433	0.747	0.012	S	4
107702	742	0.189	0.042	0.841	_	2
150151	257	0.413	0.059	0.588	_	5
49748	386	0.015	0.906	0.209	S	2
150399	588	0.123	0.817	0.031	S	2
36985	453	0.051	0.294	0.694	_	4
36994	589	0.045	0.917	0.036	S	1
130948	590	0.038	0.942	0.053	S	1
36996	588	0.066	0.925	0.036	S	1
37005	203	0.044	0.261	0.726	_	3
37023	753	0.068	0.477	0.496	_	5
396825	410	0.022	0.966	0.049	S	1
37051	681	0.056	0.101	0.905	_	1
167339	723	0.081	0.047	0.918	_	1
108031	840	0.039	0.298	0.854	_	3
60306	510	0.96	0.018	0.089	M	1
94900	199	0.349	0.054	0.575	_	4
150787	929	0.174	0.491	0.328	S	5
108631	254	0.7	0.029	0.417	M	4
151004	587	0.056	0.645	0.36	S	4
408988	1,119	0.13	0.887	0.023	S	2
37467	467	0.03	0.984	0.031	S	1
131760	370	0.03	0.705	0.316	S	4
412878	510	0.049	0.888	0.057	S	1
109222	337	0.086	0.929	0.02	S	1
420841	296	0.43	0.109	0.29	M	5
167984	273	0.256	0.065	0.614	_	4
37832	314	0.294	0.736	0.057	S	3
181255	402	0.021	0.96	0.068	S	1
37882	458	0.034	0.945	0.064	S	1
168122	772	0.03	0.949	0.05	S	1
132435	597	0.138	0.364	0.28	S	5
424941	446	0.025	0.965	0.038	S	1
69748	2,350	0.114	0.907	0.026	S	2
38169	565	0.021	0.958	0.058	S	1
38189	796	0.115	0.147	0.695	_	3
109757	566	0.908	0.033	0.094	M	1
38208	806	0.057	0.966	0.015	S	1
50823	892	0.053	0.066	0.95	_	1
38229	528	0.123	0.923	0.014	S	2
69931	375	0.044	0.99	0.027	S	1
61232	400	0.265	0.256	0.365	_	5
38397	558	0.019	0.947	0.08	S	1
133171	563	0.194	0.78	0.019	S	3
38406	253	0.17	0.863	0.026	S	2
38407	253	0.12	0.822	0.058	S	2
110758	322	0.172	0.383	0.274	S	5
152728	384	0.062	0.798	0.143	S	2
110978	740	0.279	0.141	0.526	_	4
168656	547	0.041	0.97	0.032	S	1
111162	528	0.041	0.316	0.851	_	3
434943	523	0.146	0.921	0.011	S	2
61366	344	0.068	0.951	0.018	S	1
38562	579	0.048	0.961	0.032	S	1
61437	560	0.053	0.951	0.021	S	1
111196	150	0.118	0.118	0.85	_	2
38632	752	0.11	0.714	0.134	S	3
38673	589	0.045	0.9	0.09	S	1
38796	890	0.051	0.889	0.068	S	1
111761	408	0.065	0.959	0.023	S	1
441800	339	0.362	0.212	0.32	M	5
169026	714	0.062	0.987	0.015	S	1
153331	307	0.041	0.53	0.552	_	5
153350	869	0.293	0.063	0.66	_	4
111954	463	0.02	0.981	0.054	S	1
61758	254	0.03	0.337	0.811	_	3
51514	257	0.043	0.288	0.734	_	3
111348	534	0.03	0.978	0.038	S	1
169283	531	0.071	0.944	0.017	S	1
39120	460	0.039	0.139	0.91	_	2
153798	467	0.061	0.158	0.896	_	2
182393	468	0.038	0.133	0.935	_	1
112304	545	0.154	0.113	0.64	_	3
39290	605	0.079	0.129	0.844	_	2
214618	503	0.081	0.056	0.926	_	1
39296	269	0.061	0.915	0.031	S	1
39375	890	0.055	0.865	0.069	S	2
51842	588	0.112	0.122	0.793	_	2
51888	475	0.314	0.663	0.039	S	4
182705	511	0.053	0.883	0.083	S	1
219817	369	0.317	0.328	0.142	S	5
219843	336	0.45	0.866	0.004	S	3
182872	668	0.034	0.956	0.057	S	1
39816	471	0.021	0.96	0.064	S	1
227734	617	0.118	0.049	0.865	_	2

Abbreviation: cTP, chloroplast transit peptide; Len, Sequence length; Loc, prediction of localization; M, Mitochondrion; RC, Reliability class; S, secretory pathway; SP, signal peptide.
